# Direct Visualization of CHIP-Mediated Degradation of Alpha-Synuclein *In Vivo*: Implications for PD Therapeutics

**DOI:** 10.1371/journal.pone.0092098

**Published:** 2014-03-24

**Authors:** Hemi Dimant, Liya Zhu, Laura N. Kibuuka, Zhanyun Fan, Bradley T. Hyman, Pamela J. McLean

**Affiliations:** 1 MassGeneral Institute for Neurodegenerative Disease, Department of Neurology, Massachusetts General Hospital, Harvard Medical School, Charlestown, Massachusetts, United States of America; 2 Department of Neuroscience, Mayo Clinic, Jacksonville, Florida, United States of America; University of Florida, United States of America

## Abstract

Parkinson's disease is a neurodegenerative disorder characterized by Lewy bodies, a pathological hallmark comprised mostly of aggregated alpha synuclein. Accumulating evidence demonstrates the association of smaller oligomeric aggregates to disease etiology and many therapeutic approaches are aimed at inhibiting and reducing the aggregation process. Molecular chaperones and co-chaperones play a key role in protein homeostasis and have potential as therapeutics to inhibit alpha synuclein associated toxicity. Here we use a gene therapy approach to evaluate the applicability of the Hsp70 co-chaperone CHIP (C-terminal Hsp70 interacting protein) as a therapeutic candidate and examine its direct effect on alpha synuclein aggregates in vivo. Utilizing a novel viral vector mediated rat model to directly detect alpha synuclein aggregates, we show that CHIP can mediate the degradation of alpha synuclein aggregates in vivo. However, our studies also reveal that CHIP may potentially degrade tyrosine hydroxylase which would compromise the applicability of CHIP as a therapeutic approach for Parkinson's disease.

## Introduction

Many neurodegenerative diseases share the common characteristic of protein misfolding and consequent aggregation leading to cell death. In the case of alpha-synucleinopathies including Parkinson's disease (PD), many lines of evidence demonstrate a strong association between alpha synuclein (α-syn) aggregation and neurodegeneration [1], [2], [3], [4]. Although the identity of toxic α-syn aggregates remains to be determined, many disease modifying therapeutic approaches aim to inhibit and reduce the aggregation process [5]. The physiological role of molecular chaperones and co-chaperones in protein homeostasis offers a promising therapeutic approach for synucleinopathies, with multiple studies demonstrating that a reduction in α-syn protein levels reduces oligomerization and rescues neurotoxicity [6], [7]. Having previously demonstrated the ability of the co-chaperone C-terminus Hsp70 interacting protein (CHIP) to target toxic α-syn aggregates for degradation *in vitro* [8], [9], we now describe the modulation of α-syn aggregates by CHIP *in vivo* in a novel viral-vector mediated rat model [10]. In this model the direct detection of α-syn aggregates using *in vivo* protein-fragment complementation facilitates the examination and quantitation of CHIP-induced modulation of α-syn aggregates *in vivo*. We demonstrate that co-expression of CHIP results in a significant reduction in α-syn aggregates *in vivo*. Somewhat surprisingly, our results also indicate that tyrosine hydroxylase may be a substrate for CHIP-mediated degradation which would preclude CHIP as a potential treatment for PD and synucleinopathies. Nonetheless, we demonstrate the direct visualization of α-syn degradation *in vivo* and validate the applicability of our animal model in examining potential therapeutic approaches that target α-syn aggregates.

## Results

We have recently described a novel rat viral vector model with the capability to directly detect and visualize α-syn aggregates *ex vivo* and *in vivo* by monitoring venusYFP fluorescence [10]. This is achieved using an *in vivo* complementation assay with targeted co-delivery of AAV-venus1-synuclein and AAV-synuclein-venus2 (AAV-V1S+ AAV-SV2) into the SNpc, which results in α-syn-induced neurodegeneration in the SNpc and reconstitution of venusYFP fluorescence along the nigrostriatal pathway. In the current study we examine if the Hsp70 co-chaperone protein CHIP can reduce α-syn oligomerization and concomitantly rescue α-syn–induced neurotoxicity *in vivo.*

**Figure 1:**
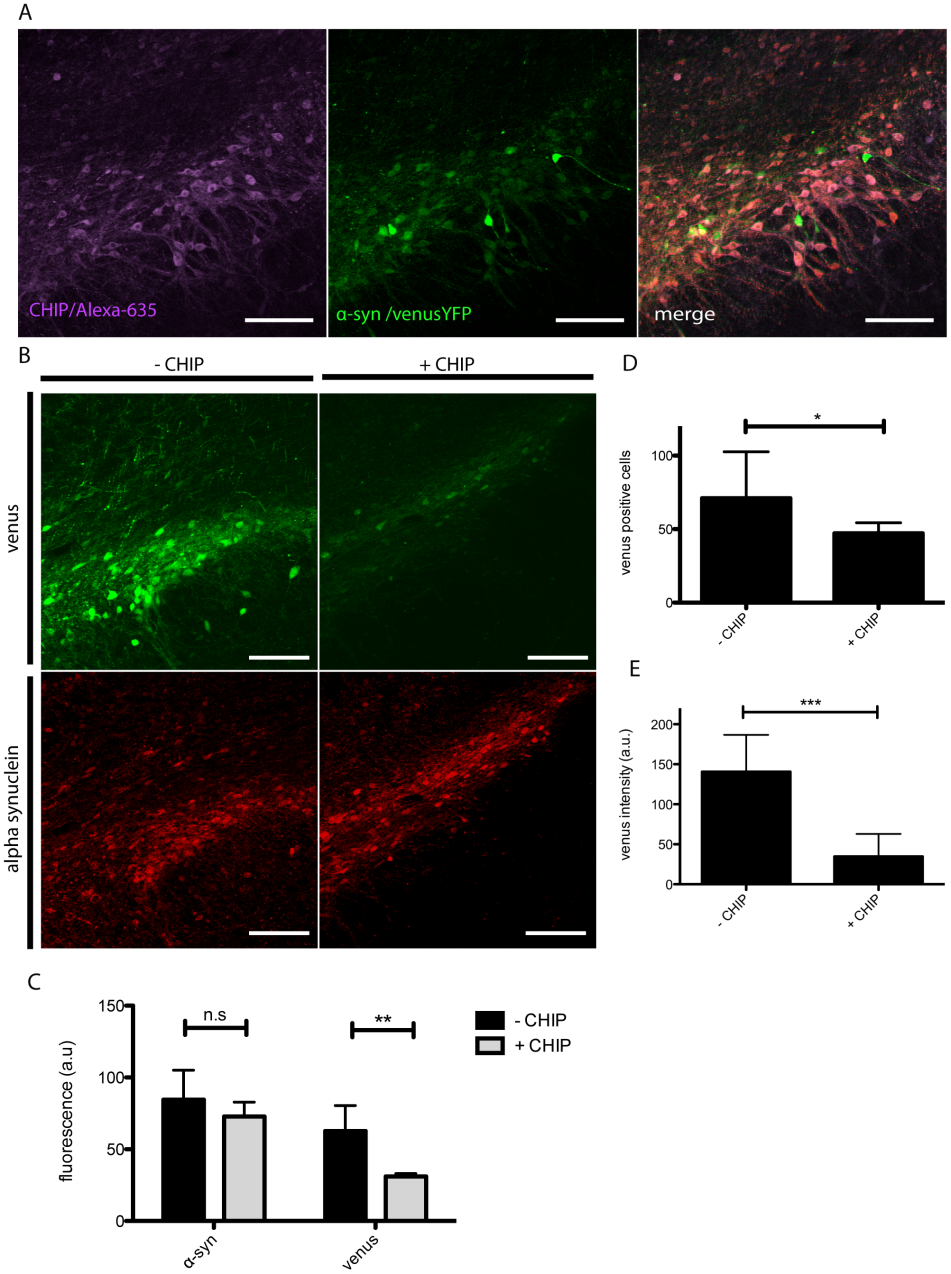
Reduced venusYFP fluorescence in the presence of CHIP. Coronal sections of the SNpc were immunostained with anti-Myc (alexa-635, purple) to evaluate co-expression of CHIP with α-syn aggregates (venusYFP, green). Images revealed extensive co-expression of CHIP and α-syn aggregates demonstrating efficient viral co-transduction (A). Coronal Sections were imaged for venusYFP fluorescence and α-syn immunostaining (red) to evaluate the level of α-syn aggregates (B). Image analysis demonstrates in syn+CHIP group (+CHIP) a significant reduction in the overall venusYFP fluorescence and no change in α-syn immunostaining compared to syn-CHIP (−CHIP)(C). Detailed image analysis shows a significant 35% reduction in the number of venusYFP positive cells in the syn+CHIP group compared to syn-CHIP (D) as well as a 4-fold reduction in venusYFP fluorescence per cell compared to control (E). Representative images are displayed. Scale bars 200 µm.

The SNpc of Sprague Dawley rats was unilaterally co-injected with AAV8-V1S, AAV8-SV2 and AAV8-myc-CHIP (syn+CHIP) or AAV8-V1S, AAV8-SV2 and empty AAV (syn-CHIP) or AAV8-myc-CHIP, AAV8-venusYFP and empty AAV (venus+CHIP) and sacrificed 8 weeks post injection. Post mortem microscopic analyses reveals the direct detection of α-syn-venusYFP in the SNpc, as described previously [10] and immunohistochemical analyses shows co-expression of CHIP with venusYFP fluorescence in the cells of the SNpc. These data indicate efficient co-transduction and co-expression of all three injected AAVs (Fig. 1A). As expected, no expression is detected contralateral to the injection site as described previously [10]. The cellular levels of α-syn and venusYFP fluorescence in cell soma and processes were examined in coronal sections of the SNpc using the human specific anti-α-syn antibody 4B12 (Fig. 1B). Interestingly, in rats co-expressing CHIP and α-syn venusYFP fluorescence was reduced by 50% compared to control animals (Fig. 1C). In the same regions of interest (ROIs), α-syn immunostaining was reduced by 15% in CHIP expressing animals compared to control animals (Fig. 1C). Detailed image analysis of the SNpc revealed a 35% reduction in the number of venusYFP positive cells (Fig. 1D) as well as a 4-fold decrease in the average fluorescence per cell in CHIP-transduced rats compared to control rats (Fig. 1E).

**Figure 2:**
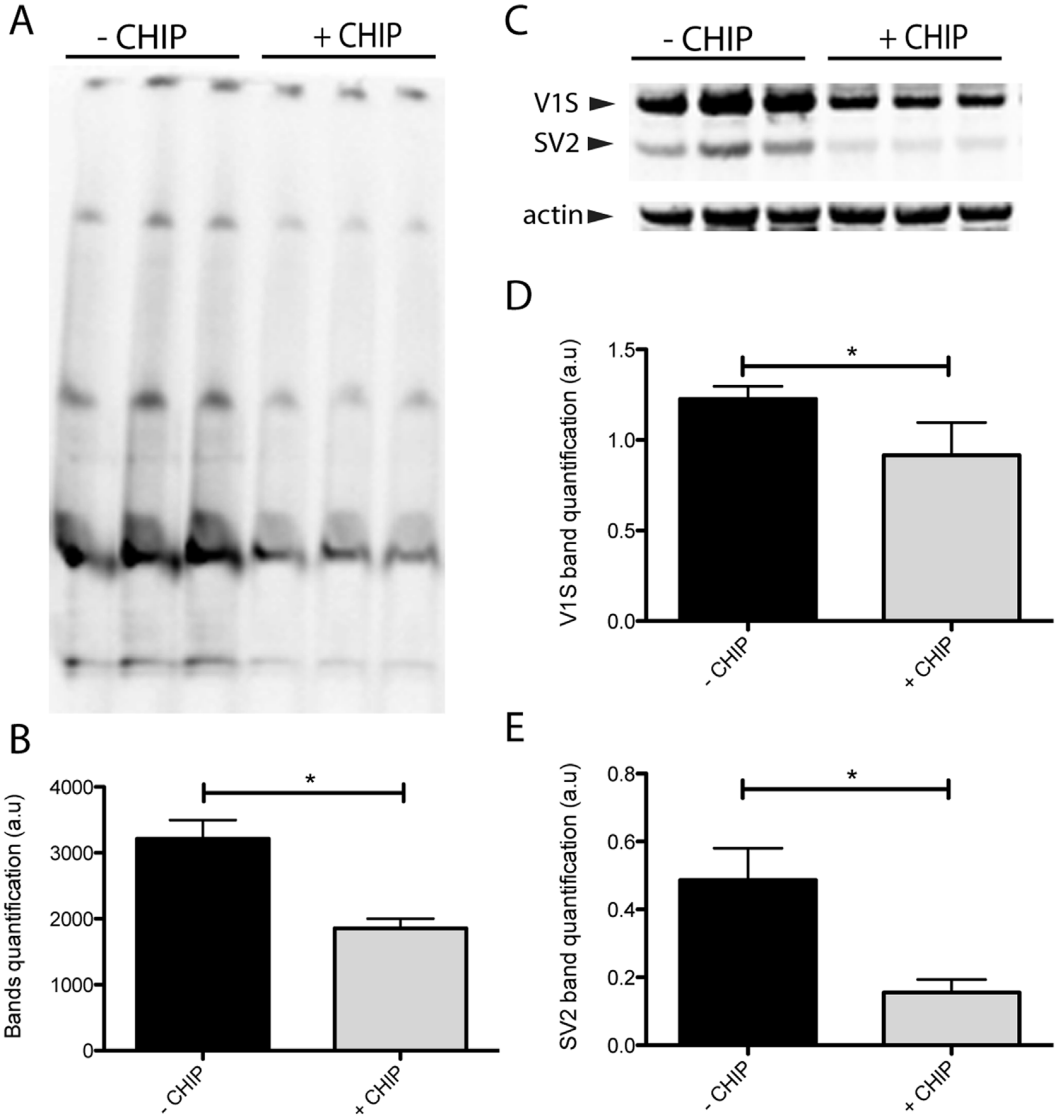
CHIP reduces the amount of human α-syn in the striatum. Striatal homogenates were biochemically analyzed for α-syn levels. Higher molecular weight α-syn species are visible in native-PAGE (A). Band quantification revealed a 43% reduction in α-syn aggregates measured in the syn+CHIP group (+CHIP) compared to the syn-CHIP group (−CHIP) (B). Denatured samples were also run on SDS-PAGE to evaluate the total amount of α-syn. The higher band at ∼31.3 KD and lower band at ∼22.4 KD detected with anti-α-syn correspond to V1S and SV2 respectively (C). A 26% and a 69% reduction were measured in both V1S and SV2 respectively in animals expressing CHIP (D and E).

To determine if CHIP-mediated degradation of α-syn aggregates is responsible for the observed reduction in venusYFP fluorescence we performed immunoblot analyses of striatal homogenates from the same animals. We have previously shown that α-syn aggregates can be detected in the striatum following injection of AAV-V1S and AAV-SV2 into SNpc. Here, striatal homogenates were analyzed using both SDS-PAGE and native PAGE to determine the level of α-syn aggregates present (Fig. 2). In order to exclude endogenous α-syn from our analyses, western blots were incubated with human specific anti α-syn antibody 4B12. High molecular weight species were detected by native PAGE confirming the formation of aggregates in this model (Fig. 2A). Furthermore, densitometry revealed a reduction in high molecular weight α-syn species in syn+CHIP animals (n = 6) compared to syn-CHIP animals (n = 3) indicating a reduction in α-syn aggregates (Fig. 2B). The total amount of human α-syn was quantified using SDS-PAGE (Fig. 2C). V1S (31 kDa) and SV2 (22 kDa) were reduced by 26% (Fig. 2D) and 69% (Fig. 2E) respectively in CHIP animals. .

**Figure 3:**
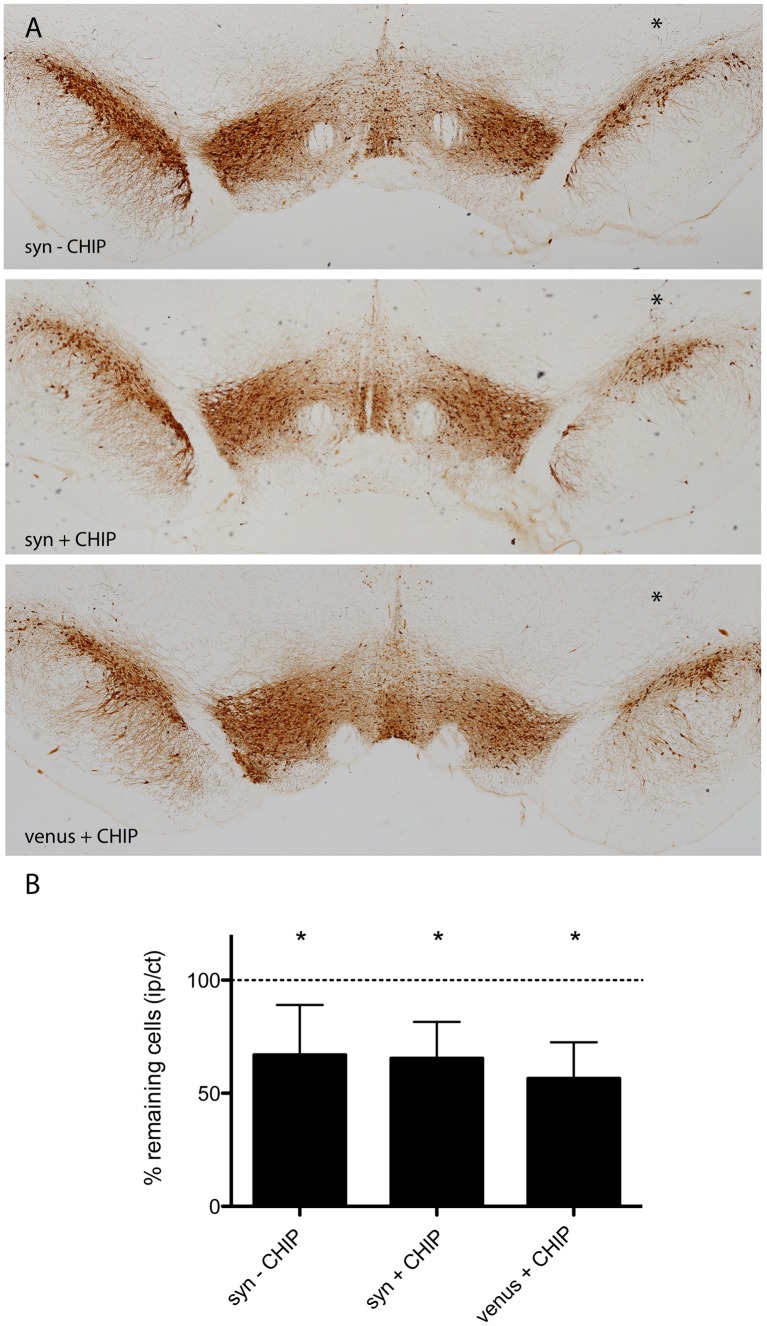
TH cell loss in the presence of CHIP. Unbiased blinded stereological analysis of TH immunopositive cells in coronal sections across the SNpc was performed using DAB (A). Cell loss is determined by the ratio of TH positive cells ipsilateral (right, asterisk) to contralateral (left). A 34% lesion and a 35% lesion were measured in the syn–CHIP group (n = 10, p<0.005) and syn+CHIP group (n = 7, p<0.005) respectively (B). The venus+CHIP group expressing only venusYFP and CHIP had a 44% lesion (n = 4, p<0.05). Representative images are displayed. Scale bar 500 µm.

**Figure 4:**
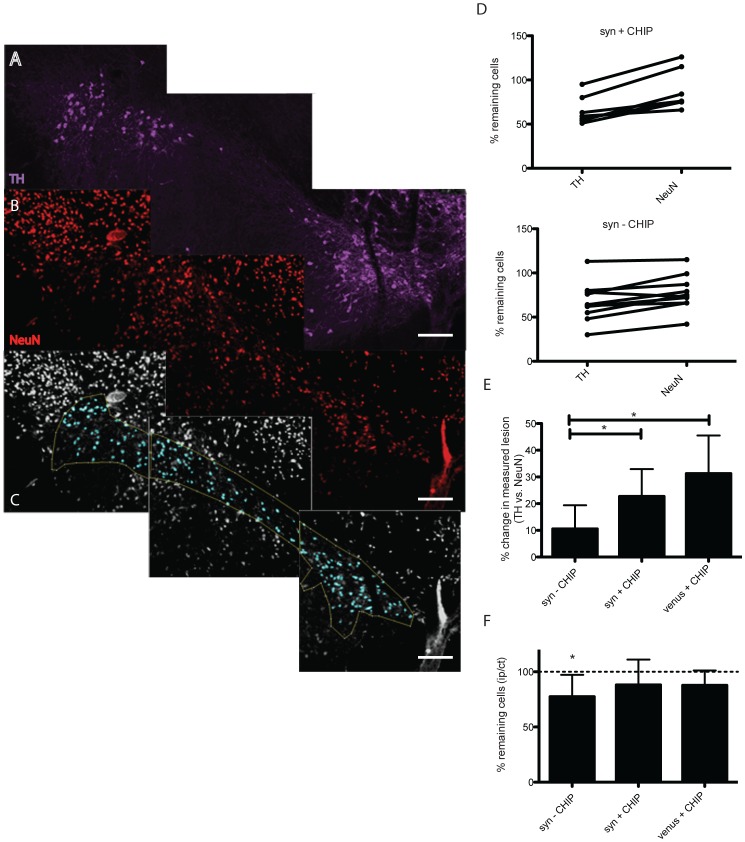
NeuN quantification in the presence of CHIP. Blinded stereological analysis of NeuN immunopositive cells in coronal sections across the SNpc was performed. Sections were co-stained with anti TH antibody (A., purple) and NeuN (B., red). A binary image was produced where the SNpc is delineated according to the TH immunostaining (C., blue). Comparison of percent cell loss per animal revealed discrepancies in the TH measured lesion compared to NeuN in the presence of CHIP (E), unlike animals not expressing CHIP (D). We found a 20–30% difference in lesions determined by NeuN to that determined by TH in the presence of CHIP (F). A significant 23% NeuN lesion was measured in the syn–CHIP group whereas no significant lesion was measured in the venus+CHIP group and syn+CHIP (F). Scale bars 200 µm. For the purpose of illustrating the image analysis conducted in this assay only representative images of CHIP animal are presented.

We have previously demonstrated α-syn-mediated neurotoxicity in rats injected with V1S+SV2 in the SNpc [10]. To determine if co-expression of CHIP is neuroprotective *in vivo* we examined neuronal survival in the SNpc of CHIP transduced rats compared to control rats 8 weeks post injection. Coronal sections of SNpc were immunostained for tyrosine hydroxylase (TH) and TH immunopositive neurons in ipsilateral and contralateral SNpc were quantified using unbiased stereology (Fig. 3A). As expected, a 34% lesion was calculated in the syn-CHIP group (n = 10). Surprisingly, a significant 35% (n = 7) lesion was observed in the syn+CHIP group despite a significant decrease in α-syn aggregation (Fig. 3B). Unexpectedly, a 44% lesion was also detected in the venus+CHIP group (n = 4) expressing full length venus and CHIP (Fig. 3B). To confirm the extent of the lesion within the SNpc we conducted secondary analyses using the neuronal marker NeuN. Coronal sections of the SNpc were co-immunostained with anti-TH antibody (Fig. 4A) and anti-NeuN antibody (Fig. 4B). Co-staining with TH and NeuN allowed us to delineate the SNpc based on TH immunostaining ipsilateral and contralateral (Fig. S1), and to then assess the number of neurons within that region using the NeuN immunostaining (Fig. 4C). We then compared cell counts of TH immunopositive cells to NeuN immunopositive cell counts for each animal (Fig. 4D & E). Recounting of NeuN immunopositive cells within the delineated SNpc revealed no neurodegeneration in animals co-expressing CHIP. Instead, our analyses revealed the difference between the lesion determined by NeuN compared to that determined by TH immunostaining (Fig. 4F). Significant neuronal loss was confirmed to persist in the syn-CHIP group (Fig. 4G).

## Discussion

Toxicity of α-syn aggregates is the focus of many disease modifying therapeutic approaches for PD. We have recently described a novel rat model enabling the direct visualization of α-syn aggregates along the nigrostriatal pathway [10]. This novel model offers a powerful tool with which to examine potential therapeutic approaches targeting α-syn aggregates and is the focus of the work presented herein. Molecular chaperones have been previously shown to degrade α-syn aggregates and thus are natural candidates in the search for disease modifying agents [6], [7]. Specifically, the co-chaperone CHIP has been shown by our laboratory to degrade and reduce α-syn aggregates *in vitro* [8], [9]. Here we used a gene therapy approach to overexpress the co-chaperone CHIP in the SNpc of model rats to determine the ability of CHIP to modify α-syn aggregates *in vivo* and rescue α-syn induced neurodegeneration. Post-mortem immunohistological analyses of rats co-injected with AAV-V1S + AAV-SV2 + AAV-CHIP (syn+CHIP) revealed substantial co-staining of venusYFP and CHIP. Since venusYFP fluorescence occurs only upon co-transduction of V1S and SV2, the observed co-expression with CHIP demonstrates the efficient co-transduction of all three viruses in neurons of the SNpc.

The significant reduction in venusYFP fluorescence within cells, as well as the reduction in the number of venusYFP positive cells following CHIP expression, is indicative of a CHIP-associated reduction of α-syn aggregates. These results also demonstrate our ability to directly detect changes in α-syn aggregate formation, thus emphasizing the advantage of our novel animal model as a tool to test potential therapeutic approaches targeting α-syn aggregates.

Further biochemical analyses showed a significant reduction in the total protein levels of α-syn as well as a reduction in α-syn aggregates which supports CHIP-mediated degradation of α-syn, in accordance with our previous studies demonstrating degradation *in vitro* [8]. However, the exact mechanism by which CHIP degrades α-syn aggregates *in vivo* remains to be determined as it was demonstrated by us and others that CHIP can target proteins for degradation via both the lysosomal and proteasomal pathways [11], [12], in both a HSP dependent and independent manner [13].

There is a possibility that the observed reduction in α-syn immunostaining is attributable to the simultaneous co-expression of three proteins. Arguing against this possibility is the differential decrease in SV2 versus V1S expression (Fig. 2D and 2E), which suggests an active degradation process, rather than a passive reduction, which would be expected to affect all proteins to the same extent. Further, the differential degradation of SV2 over V1S should be taken into consideration when comparing the level of venusYFP fluorescence to immunostaining of α-syn. Simply put, degradation of SV2 will result in reduced venusYFP fluorescence but the remaining V1S would still be detected with immunostaining. This is the most likely explanation for the significant reduction in venusYFP fluorescence observed in the SNpc compared to the non-significant reduction in α-syn staining.

In this study we have used two different cellular markers, TH and NeuN, to determine neuronal survival. In both the syn-CHIP group and the syn+CHIP group we detected a loss of TH immunopositive cells in the SNpc. Unexpectedly, we also detected a loss of TH immunopositive cells in the SNpc of the venus+CHIP group. Since this group does not overexpress α-syn, the apparent neuronal loss could not be associated with α-syn. Even though we have previously shown that overexpression of CHIP and venusYFP is non-toxic [8], [9], [10], this result could be interpreted as CHIP-mediated neurotoxicity. However the fact that CHIP has been demonstrated to be non-toxic in vitro, led us to question TH as a valid dopaminergic cell marker by which to determine neurodegeneration in the SNpc. The fact that CHIP is an E3-ubiquitin ligase with several know substrates [14], [15] raises the possibility of CHIP-mediated degradation of TH, which may appear as neuronal loss. In other words, viable dopaminergic neurons expressing CHIP would be wrongly excluded from our analysis when counting TH immunopositive cells because of CHIP-mediated TH degradation. To address this potential conflict we conducted a second stereological analysis using NeuN as a marker to determine neuronal viability in the SNpc. This approach allowed us to not only re-evaluate possible CHIP-associated cell loss but also allowed us to indirectly examine the possibility of CHIP-mediated degradation of TH. Re-analysis of NeuN immunopositive cells in the SNpc demonstrated a significantly greater number of neurons ipsilateral to the injection site. Furthermore, NeuN immunostaining revealed the absence of cell loss in both groups expressing CHIP, whereas significant neuronal loss was still apparent in the syn-CHIP group. The conflicting neuronal counts determined by TH versus NeuN in the +CHIP groups, support our original hypothesis that CHIP may target TH for degradation and may be neuroprotective. However, the lack of significant cell loss via NeuN staining in the +CHIP groups, should be interpreted with caution given the fact that cell loss was apparent when TH immunopositive cells were quantified Taken together, the immunohistochemical analysis conducted in this study is insufficient to definitively determine the exact effect of CHIP on neuronal survival despite the CHIP-mediated degradation of α-syn aggregates that was observed. Irrespective, the possibility that CHIP targets tyrosine hydroxylase for degradation precludes it as a possible therapeutic intervention for PD since TH is a rate-limiting enzyme in the conversion of the amino acid tyrosine to the neurotransmitter dopamine [16], which is depleted in PD. Although CHIP may not ultimately be a suitable therapeutic approach for PD, it is still a valid approach for other protein-misfolding diseases such as Alzheimer's disease and Huntington's disease and was previously shown to degrade protein aggregates implicated in both diseases [12], [17], [18].

Here we examine the direct delivery of a viral vector expressing the co-chaperone CHIP into the SNpc of a unique PD rat model as a gene therapy approach to reduce α-syn-induced neurotoxicity. Despite the described limitations, this work demonstrates the potential of our novel model for evaluating therapeutic candidates, which is a valuable tool currently lacking in the search for potential therapies for synucleinopathies.

## Methods

### Virus preparation

The viral vectors pAAV-CBA-VENUS1-SYNUCLEIN-WPRE (V1S) and pAAV-CBA-SYNUCLEIN-VENUS2-WPRE (SV2) were constructed by inserting the human α-syn cDNA, fused to either the N-terminus half of venusYFP (V1S) or the C-terminus half of venusYFP (SV2), into the EcoRV and NheI sites of the pAAV-CBA-WPRE vector [7]. pAAV-CBA-VenusYFP-WPRE was constructed by inserting the venusYFP cDNA into the XhoI and NheI sites of pAAV-CBA-WPRE vector. pAAV-CBA-myc-CHIP was constructed by inserting the myc-tagged CHIP cDNA into pAAV-CBA-WPRE vector. AAV serotype 8 was produced by the Harvard Gene Therapy Initiative as previously described [19].

### Injections

Sprague Dawley rats (300–350 gram) were anesthetized with intraperitoneal injection of ketamine/xylazine and placed in a stereotaxic frame. The surgical site was shaved and sterilized with betadine prior to making a 2 cm incision along the midline. The scalp was exposed and a unilateral injection targeting the SN was performed at coordinates AP −5.2, ML −2 and DV −7.4 with bregma as a point of reference. For each animal, a total volume of 3 µl of virus was injected at a rate of 0.2 µl/min using a microinjection pump and 10 µl Hamilton syringe with a 30-gauge needle. For V1S+ SV2+ CHIP group 1 µl of AAV8- V1S (8.3•10^12^ viral genome/ml), 1 µl of AAV8-SV2 (8.7•10^12^ viral genome/ml) and 1 µl of AAV8- CHIP (1.2•10^13^ viral genome/ml) were coinjected; for the V1S+ SV2- CHIP group (control), 1 µl of AAV8- V1S and 1 µl of AAV8-SV2 were coinjected. This group included several animals injected with additional 1 µl of an empty AAV8. Animals in the venusYFP+CHIP group (venus+CHIP) were injected with 1 µl of AAV8-venusYFP (1•10^12^ viral genome/ml), 1 µl of AAV8-myc-CHIP and 1 µl an empty AAV8. At the end of injection period the needle remained in place for 5 minutes before gradual removal.

All studies were performed with the approval of the Massachusetts General Hospital Animal Care and Use Committee and in compliance with the National Institute of Health guidelines for the use of experimental animals.

### Tissue processing

At 8 weeks post-injection, rats were deeply anesthetized and transcardially perfused with 0.01 M phosphate buffered saline (PBS, pH7.4, Sigma) followed by brain extraction. The striatum was freshly dissected for biochemical analysis prior to post fixation of the midbrain, which allowed us to conduct biochemical and histological analysis from the same animal. Brains were post-fixed for 24–72 hours and transferred to a solution of 30% sucrose in PBS. Frozen brains were sectioned at 40 µm on a sliding microtome and kept in a cryoprotectant solution (30% sucrose, 30% ethylene glycol in PBS). Frozen striatum were thawed, homogenized in PBS (1∶4 weight/volume) and kept at −20°C.

### Immunostaining

Free-floating coronal sections were washed overnight in PBS to remove cryoprotectant. Unless otherwise stated, all steps were performed at room temperature with three 10 min washes in PBS-TX (PBS with 0.3% triton X-100) between each step. For diaminobenzidine (DAB) staining sections were treated with 10% methanol and 3% H_2_O_2_ to inhibit endogenous peroxidases, permeabilized in PBS-TX for 30 min and blocked in 5% NGS in PBS-TX for 30 min. For non-fluorescence TH staining, sections were incubated with primary antibody rabbit anti-TH (1∶10,000, Millipore) overnight at 4°C followed by secondary goat anti rabbit-biotin (1∶200 Jackson ImmunoResearch) for 1 hour at room temperature. Sections were incubated with DAB (Vector laboratories) to visualize TH positive cells, rinsed in PBS, mounted on superfrost slides (Fisher scientific) and coverslipped (Permount, Sigma).

For immunofluorescence, sections were incubated with mouse anti α-syn (4B12 1∶1000, Signet) or mouse anti-NeuN (1∶500, Millipore) together with rabbit anti-TH (1∶10,000, Millipore) or mouse anti c-myc clone 9E10 (1∶1000, Abcam) overnight at 4°C. Sections immunostained with anti-TH and anti-NeuN were then incubated with secondary goat anti rabbit-biotin (1∶200, Jackson ImmunoResearch) and goat anti mouse-cy3 (1∶500, Jackson ImmunoResearch) for 1 hour at room temperature followed by streptavidin-alexa635 (1∶200, Life Sciences) for 1 hour at room temperature. Sections immunostained with either anti-c-myc or anti-α-syn were incubated with goat anti rabbit-biotin or goat anti mouse-biotin respectively (1∶200, Jackson ImmunoResearch) for 1 hour at room temperature followed by streptavidin-alexa635 or streptavidin-alexa555 respectively (1∶200, Life Sciences) for 1 hour at room temperature. Sections were mounted onto superfrost slides, coverslipped with vectashield (Vector laboratories) and kept at 4°C.

### Western Blot

Protein concentration of striatal homogenates was determined using BCA kit (Pierce) and 25 µg proteins were loaded onto polyacrylamide gels (Nupage). For denatured Western blot, protein samples were loaded onto Nupage 4–12% SDS-PAGE gels and run according to the manufacturer instructions. Gels were transferred onto 0.4 µm nitrocellulose membranes. For native gels, non-denatured protein samples were loaded onto 4–16% Bis-Tris native gels (Nupage) and run at 150V for 2 hours on ice. Native gels were transferred onto PVDF membranes using 25V for 1 hour on ice. Following transfer, membranes were fixed in 8% acetic acid for 15 minutes and rinsed with water. Both PVDF and nitrocellulose membranes were incubated with mouse anti-α-syn (4B12 1∶1000, Signet) and rabbit anti-β-actin in blocking solution (Li-Cor) overnight at 4°C. Membranes were then incubated with goat anti mouse-IR800 and goat anti rabbit-IR680 for 1 hour in blocking solution (Li-Cor) prior to imaging on an Odyssey imager.

### Microscopy, stereology and image analysis

Fluorescence images were obtained on either a Zeiss LSM510 META confocal microscope with X20 magnification for fluorescence imaging or an Olympus BX51 microscope for DAB imaging. At least 8 coronal sections per animal throughout the SNpc were analyzed to determine tyrosine hydroxylase cell loss by conducting unbiased stereology cell counting using the Olympus CAST stereology software. An average of 300 cells in the SNpc were analyzed for venusYFP fluorescence. For NeuN lesion analysis, coronal sections were co-stained with anti TH and anti NeuN antibodies. The SNpc was outlined using Fiji (NIH) according to TH staining and was saved as a region of interest. The amount of NeuN positive neurons only within that region was calculated using Fiji's particle analysis feature.

### Statistics

Mann Whitney test was performed for all data sets. One-tail t-test analysis was performed for neuronal lesion analysis presented in Figures 3 and 4. Statistical analysis was performed using GraphPad Prism software.

## Supporting Information

Figure S1 **Tissue staining and image analysis.** To count NeuN positive cells within the SNpc, coronal sections were co-immmunostained with anti-TH antibody (purple) and anti-NeuN (red). Merged image of TH and NeuN immunopoitive cells (merge) illustrate the region in which NeuN counting was conducted according to TH staining. The SNpc was delineated ipsilateral and contralateral and defined as a region of interest according to the TH immunostaining, which was then superimposed onto the NeuN image to allow particle analysis. NeuN positive cells were counted within the defined region across the SNpc of each animal. For the purpose of illustrating the image analysis conducted in this assay a representative images of a CHIP injected animal is presented.
(TIF)

## References

[1] Irizarry MC, Growdon W, Gomez-Isla T, Newell K, George JM, et al (1998) Nigral and cortical Lewy bodies and dystrophic nigral neurites in Parkinson's disease and cortical Lewy body disease contain alpha-synuclein immunoreactivity. J Neuropathol Exp Neurol 57: 334–337. 9600226 10.1097/00005072-199804000-00005

[2] Chartier-Harlin MC, Kachergus J, Roumier C, Mouroux V, Douay X, et al (2004) Alpha-synuclein locus duplication as a cause of familial Parkinson's disease. Lancet 364: 1167–1169. 15451224 10.1016/S0140-6736(04)17103-1

[3] Winner B, Jappelli R, Maji SK, Desplats PA, Boyer L, et al (2011) In vivo demonstration that alpha-synuclein oligomers are toxic. Proc Natl Acad Sci U S A 108: 4194–4199. 21325059 10.1073/pnas.1100976108 PMC3053976

[4] Spillantini MG, Crowther RA, Jakes R, Hasegawa M, Goedert M (1998) alpha-Synuclein in filamentous inclusions of Lewy bodies from Parkinson's disease and dementia with lewy bodies. Proc Natl Acad Sci U S A 95: 6469–6473. 9600990 10.1073/pnas.95.11.6469 PMC27806

[5] Kalia SK, Kalia LV, McLean PJ (2010) Molecular chaperones as rational drug targets for Parkinson's disease therapeutics. CNS Neurol Disord Drug Targets 9: 741–753. 20942788 10.2174/187152710793237386 PMC3364514

[6] Outeiro TF, Klucken J, Strathearn KE, Liu F, Nguyen P, et al (2006) Small heat shock proteins protect against alpha-synuclein-induced toxicity and aggregation. Biochem Biophys Res Commun 351: 631–638. 17081499 10.1016/j.bbrc.2006.10.085 PMC1934426

[7] Danzer KM, Ruf WP, Putcha P, Joyner D, Hashimoto T, et al (2011) Heat-shock protein 70 modulates toxic extracellular alpha-synuclein oligomers and rescues trans-synaptic toxicity. FASEB J 25: 326–336. 20876215 10.1096/fj.10-164624 PMC3005424

[8] Tetzlaff JE, Putcha P, Outeiro TF, Ivanov A, Berezovska O, et al (2008) CHIP targets toxic alpha-Synuclein oligomers for degradation. J Biol Chem 283: 17962–17968. 18436529 10.1074/jbc.M802283200 PMC2936239

[9] Kalia LV, Kalia SK, Chau H, Lozano AM, Hyman BT, et al (2011) Ubiquitinylation of alpha-synuclein by carboxyl terminus Hsp70-interacting protein (CHIP) is regulated by Bcl-2-associated athanogene 5 (BAG5). PLoS One 6: e14695. 21358815 10.1371/journal.pone.0014695 PMC3040167

[10] Dimant H, Kalia S, Kalia L, Zhu L, Kibuuka L, et al (2013) Direct detection of alpha synuclein oligomers in vivo. Acta Neuropathologica Communications 1: 6. 24252244 10.1186/2051-5960-1-6 PMC3776213

[11] Shin Y, Klucken J, Patterson C, Hyman BT, McLean PJ (2005) The co-chaperone carboxyl terminus of Hsp70-interacting protein (CHIP) mediates alpha-synuclein degradation decisions between proteasomal and lysosomal pathways. J Biol Chem 280: 23727–23734. 15845543 10.1074/jbc.M503326200

[12] Sahara N, Murayama M, Mizoroki T, Urushitani M, Imai Y, et al (2005) In vivo evidence of CHIP up-regulation attenuating tau aggregation. J Neurochem 94: 1254–1263. 16111477 10.1111/j.1471-4159.2005.03272.x

[13] Wickner S, Maurizi MR, Gottesman S (1999) Posttranslational quality control: folding, refolding, and degrading proteins. Science 286: 1888–1893. 10583944 10.1126/science.286.5446.1888

[14] Woo CH, Le NT, Shishido T, Chang E, Lee H, et al (2010) Novel role of C terminus of Hsc70-interacting protein (CHIP) ubiquitin ligase on inhibiting cardiac apoptosis and dysfunction via regulating ERK5-mediated degradation of inducible cAMP early repressor. FASEB J 24: 4917–4928. 20724525 10.1096/fj.10-162636 PMC2992371

[15] Hwang JR, Zhang C, Patterson C (2005) C-terminus of heat shock protein 70-interacting protein facilitates degradation of apoptosis signal-regulating kinase 1 and inhibits apoptosis signal-regulating kinase 1-dependent apoptosis. Cell Stress Chaperones 10: 147–156. 16038411 10.1379/CSC-90R.1 PMC1176473

[16] Daubner SC, Le T, Wang S (2011) Tyrosine hydroxylase and regulation of dopamine synthesis. Arch Biochem Biophys 508: 1–12. 21176768 10.1016/j.abb.2010.12.017 PMC3065393

[17] Miller VM, Nelson RF, Gouvion CM, Williams A, Rodriguez-Lebron E, et al (2005) CHIP suppresses polyglutamine aggregation and toxicity in vitro and in vivo. J Neurosci 25: 9152–9161. 16207874 10.1523/JNEUROSCI.3001-05.2005 PMC6725774

[18] Petrucelli L, Dickson D, Kehoe K, Taylor J, Snyder H, et al (2004) CHIP and Hsp70 regulate tau ubiquitination, degradation and aggregation. Hum Mol Genet 13: 703–714. 14962978 10.1093/hmg/ddh083

[19] McFarland NR, Fan Z, Lee JS, Sena-Esteves M, Stern EA, et al. (2007) Comparison of adeno-associated viral serotypes for gene delivery to the nigrostriatal system. Society for Neuroscience. San Fancisco, CA.

