# Supplementary figures and images for: Direct Visualization of CHIP-Mediated Degradation of Alpha-Synuclein In Vivo: Implications for PD Therapeutics
Source: PLoS One. 2014 Mar 24;9(3):e92098. doi: 10.1371/journal.pone.0092098 (PMC3963877; doi:10.1371/journal.pone.0092098)

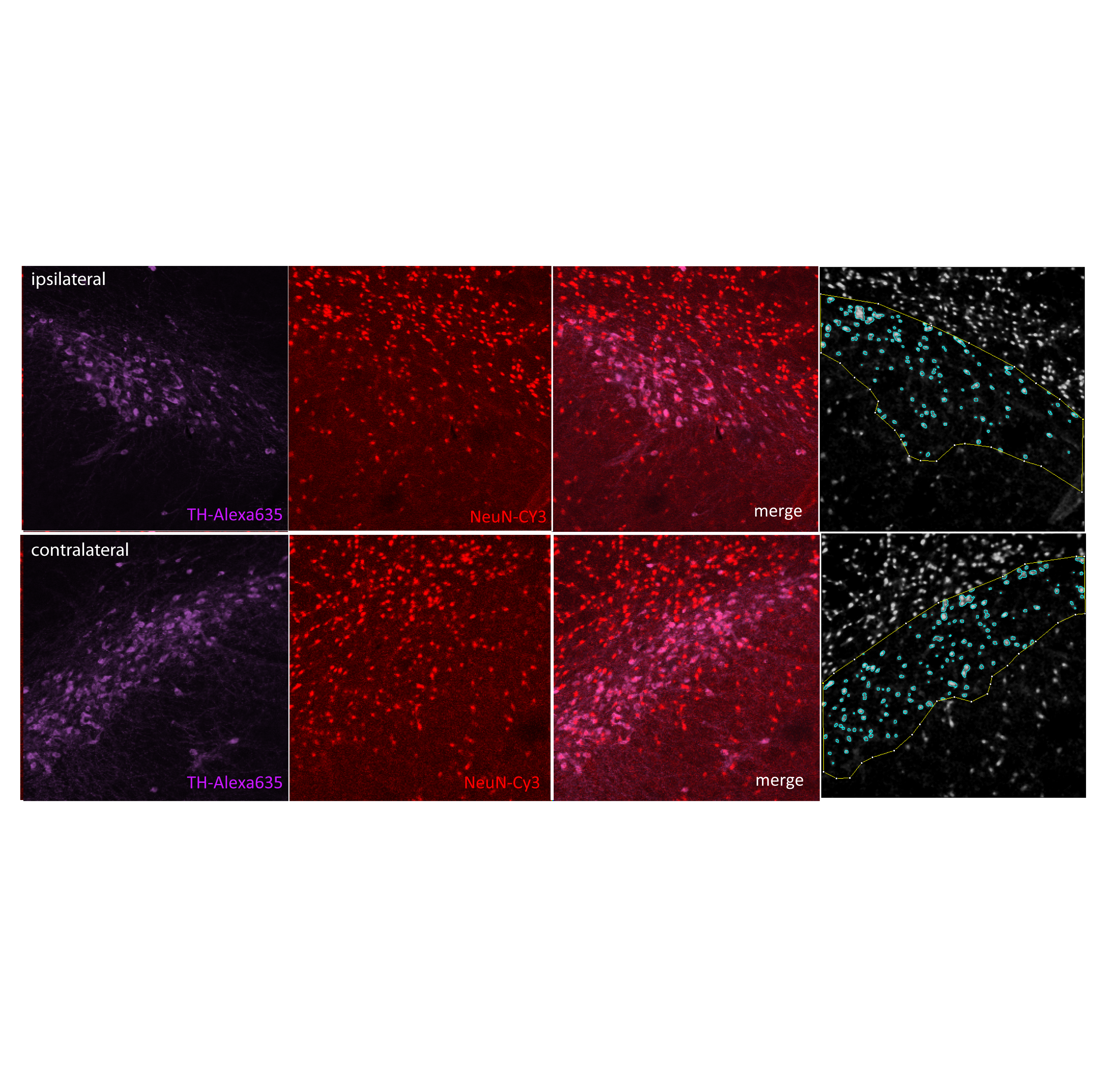

Supplement: Figure S1 — Tissue staining and image analysis. To count NeuN positive cells within the SNpc, coronal sections were co-immmunostained with anti-TH antibody (purple) and anti-NeuN (red). Merged image of TH and NeuN immunopoitive cells (merge) illustrate the region in which NeuN counting was conducted according to TH staining. The SNpc was delineated ipsilateral and contralateral and defined as a region of interest according to the TH immunostaining, which was then superimposed onto the NeuN image to allow particle analysis. NeuN positive cells were counted within the defined region across the SNpc of each animal. For the purpose of illustrating the image analysis conducted in this assay a representative images of a CHIP injected animal is presented. (TIF) [file pone.0092098.s001.tif]
